# The causes of infertility in women presenting to gynaecology clinics in Harare, Zimbabwe; a cross sectional study

**DOI:** 10.1186/s40738-020-00093-0

**Published:** 2021-01-05

**Authors:** Mugove G. Madziyire, Thulani L. Magwali, Vasco Chikwasha, Tinovimba Mhlanga

**Affiliations:** 1 Department of Obstetrics and Gynaecology, University of Zimbabwe College of Health Sciences, Harare, Zimbabwe; 2 Department of Community Medicine, University of Zimbabwe College of Health Sciences, Harare, Zimbabwe

**Keywords:** Causes of infertility, Subfertility, Aetiology of infertility, Infertility outcomes

## Abstract

**Background:**

Infertility affects 48.5 million couples globally. It is defined clinically as failure to conceive after 12 months or more of regular unprotected sexual intercourse. The contribution of various aetiological factors to infertility differs per population. The causes of infertility have not been assessed in Zimbabwe. Our objectives were to determine the reproductive characteristics, causes and outcomes of women presenting for infertility care.

**Methods:**

A retrospective and prospective study of women who had not conceived within a year of having unprotected intercourse presenting in private and public facilities in Harare was done. A diagnosis was made based on the history, examination and results whenever these were deemed sufficient. Data was analysed using STATA SE/15. A total of 216 women were recruited.

**Results:**

Of the 216 women recruited, two thirds (144) of them had primary infertility. The overall period of infertility ranged from 1 to 21 years with an average of 5.6 ± 4.7 years whilst 98 (45.4%) of the couples had experienced 2–4 years of infertility and 94 (43.5%) had experience 5 or more years of infertility. About 1 in 5 of the women had irregular menstrual cycles with 10 of them having experienced amenorrhoea of at least 1 year. Almost half of the participants (49%) were overweight or obese. The most common cause for infertility was ‘unexplained’ in 22% of the women followed by tubal blockage in 20%, male factor in 19% and anovulation in 16%. Of the 49 (22.7%) women who conceived 21(9.7%) had a live birth while 23 (10.7%) had an ongoing pregnancy at the end of follow up. Thirty-seven (17.1%) had Assisted Reproduction Techniques (ART) in the form of Invitro-fertilisation/Intracytoplasmic Sperm Injection (IVF/ICSI) or Intra-Uterine Insemination (IUI). Assisted Reproduction was significantly associated with conception.

**Conclusion:**

Most women present when chances of natural spontaneous conception are considerably reduced. This study shows an almost equal contribution between tubal blockage, male factor and unexplained infertility. Almost half of the causes are female factors constituted by tubal blockage, anovulation and a mixture of the two. Improved access to ART will result in improved pregnancy rates. Programs should target comprehensive assessment of both partners and offer ART.

**Supplementary Information:**

The online version contains supplementary material available at 10.1186/s40738-020-00093-0.

## Plain English summary

Infertility which is defined clinically as failure to conceive after 12 months or more of regular unprotected sexual intercourse affects approximately 48.5 million couples globally. Contribution of common aetiological factors to its burden differs per population. The causes of infertility and outcomes of investigations and treatment have not been assessed in Zimbabwe. We aimed to determine the reproductive characteristics, causes of infertility and outcomes in women presenting with infertility in public and private gynaecology clinics in Harare. Recruitment and follow up was from the 5th of June 2019 to the 30th of April 2020.

Of the 216 women recruited, just over half of them were aged 30–39 years and had experienced an average of 5.6 years of infertility with 94 of them having experienced more than 5 years on infertility. Two out of three women had never fallen pregnant before. Half of them were overweight or obese. Just less than half of the women had their male partners tested for sperm dysfunction and 63% of tested men showed varying degrees of sperm dysfunction. Cause of Infertility was ‘unexplained’, tubal blockage and male factor in 1out 5 women respectively. Of the 49 women who fell pregnant, 21 of them delivered a live birth by the end of follow up. Thirty-seven women had Assisted Reproduction and this was significantly associated with conception.

In conclusion, most women present when chances of natural spontaneous conception are considerably reduced and there is almost an equal contribution between tubal blockage, male and unexplained factors to infertility.

## Introduction

Infertility is a worldwide problem mainly affecting sub-Saharan Africa. The World health Organisation (WHO) recognizes it as a major public health problem [1]. Infertility is involuntary childlessness and is either primary if conception has never been achieved or secondary if conception has been experienced before. WHO clinically defines infertility as a disease of the reproductive system characterised by failure to achieve a clinical pregnancy after 12 months or more of regular unprotected sexual intercourse. The prevalence of infertility is variable depending on the definition (clinical – 1 year, epidemiological – 2 years, demographic – 5 years) and on whether the outcome is pregnancy or births [2, 3]. The latest estimate of the prevalence of infertility globally using the demographic definition and live birth as an outcome estimated that 48.5 million couples were affected by infertility. Prevalence of primary infertility was 1.9% and secondary infertility 10.5% [3].WHO estimates that these figures go up 2.5 fold using an epidemiological definition of infertility [1]. The prevalence of infertility using a clinical definition and conception as an outcome is much higher as more couples conceive naturally with progression of time. Identifying clinical infertility allows earlier assessment of affected women, however up to 15% of normal couples might fail to conceive just by chance in the first year of attempting [4]. The contribution of male and female factors is about 40% each. For women, ovulatory failure is the commonest cause (25%), followed by tubal blockage (20%). For men the commonest cause is sperm defects or dysfunction (30–40%). Unexplained infertility may be as high as 25% [5]. Prospects of pregnancy in unexplained infertility are good for women younger than 35 years and when the duration of infertility is < 2 years [4]. The contribution of various infertility aetiological factors differs per population.

There is limited access to infertility care in the developing world [6]. No study has analysed the contribution of sperm dysfunction, tubal blockage, uterine abnormalities, ovulatory dysfunction and unexplained infertility in Zimbabwe. Likewise, the number of patients with a conclusive diagnosis and those who achieve conception is presumed to be low. Understanding causes of infertility amongst women will help clinicians to focus management to options that are relevant and cost effective.

## Methods

We aimed to explore the contribution of known aetiological factors to infertility in women accessing infertility care in Harare and the adjoining city of Chitungwiza. Harare the capital city of Zimbabwe has a population of about 1.5 million [7]. Chitungwiza city which is 30 km from Harare has a population of 1.2 million [8]. Both cities are serviced by three tertiary hospitals which offer specialist gynaecology services. There are several private gynaecology clinics in the cities. A retrospective and prospective study of women who had not conceived within a year of having unprotected intercourse presenting in private and public facilities in Harare and Chitungwiza was done. The decision to retrospectively recruit in the private sector was based on the fact that records tend to be more complete as compared to the public sector. At commencement of the study, we had hoped to enrol equal numbers in both the public and private sector clinic but however, a crippling doctors’ strike and later the Covid-19 pandemic disturbed accrual in the public sector. Private sector recruitment was not affected as sampling was retrospective. Women who could not afford investigations such as hysterosalpingogram (HSG), laparoscope and dye, ovulation test and semen analyses for their partners were assisted with money to pay. More effort for the male partner to have a semen analysis was made when there was no obvious female factor identified. Where female and male assessment was normal, the cause of infertility would be classified as ‘unexplained infertility’. As such the women whose partners were not tested had to have an identifiable cause of infertility deduced from history, examination or investigations before they could qualify for inclusion. Nurses working in the gynaecology out–patient department in each hospital and all gynaecologists working in private and public facilities were notified about the study. Research midwives identified any patients with a diagnosis of infertility presenting at the public hospitals daily during the study period and consented them. The principal investigator and a research nurse from each participating hospital administered a questionnaire to all consenting participants and additional information was obtained from their hospital cards, laboratory and radiological reports. Consenting private gynaecologists were asked to retrieve records on infertile patients under their care for the previous 2 years and a questionnaire completed with data similar to what was being sought in the prospective arm of the study. The records from private had to be complete such that the cause of infertility had been established and having at least 6 months of follow up since diagnosis. The records were conveniently sampled starting with the most recent ones aiming to a maximum of 20 records from one clinic. Women with bilateral tubal blockage on either HSG or laparoscope and dye studies were classified as having tubal blockage. If the male partner had abnormalities in sperm concentration, motility or morphology the woman was classified as having male factor infertility or as male/female factor if there was a co-existent female cause of infertility. All women with regular cycles were considered ovulating whereas women with irregular infrequent scanty and sometimes absent cycles were considered anovulatory [9]. Women with classic diagnosis of Polycystic ovary syndrome (PCOS), diminished ovarian reserve and low luteal phase progesterone were also considered anovulatory [10]. Recruitment and follow up of participants in the prospective sample was from the 5th of June 2019 to the 30th of April 2020. At least 6 months of follow up was allowed from the time the definitive diagnosis was made. Information on their demographic profile, period of attempting to conceive, medical history and examination, prior investigations and current investigations was obtained on enrolment and follow up. A final diagnosis was made based on the history, examination and results. Some diagnoses were already apparent at recruitment depending on how conclusive prior history, examination and investigations were while others became apparent after further investigations and follow up.

The sample size calculated using the single proportion formula based on a study which had assessed causes of infertility in Bauchi district of Northern Nigeria was 205 [11]. Overall, 216 women were enrolled. Data was collected using a questionnaire and captured into Redcap software [12] and exported to STATA/SE 15 [10] for analysis. Descriptive summary statistics were reported as frequencies and percentages for categorical data and means and standard deviations for continuous normally distributed data. Tests for association were conducted using the chi-square test for categorical variables. Where sample size was small in some cells, the Fisher’s exact test was used. Missing data was recorded in tables as a variable choice labelled ‘not stated’/ ‘not recorded’/ ‘not done’/ ‘not weighed’.

A total of 216 women were recruited prospectively from the public sector (22%) and retrospectively from the private sector (78%) hospitals respectively in Harare, Zimbabwe.

Ethical clearance was granted by the Joint Research Ethics Committee for the University of Zimbabwe, College of Health Sciences and Group of Parirenyatwa (JREC), Harare hospital ethics committee, Chitungwiza hospital ethics committee and the Medical Research Council of Zimbabwe (MRCZ).

## Results

Of the 216 women recruited 54.2% were in the 30–39 age category. The majority (92.6%) of the participants were married. Table 1 shows the participants’ demographic profile.

**Table 1:** Demographic profile of participants

Characteristic	Frequency (%)
Source	
*Public*	47 (21.8)
*Private*	169 (78.2)
Age group (years)	
*< 30*	67 (31.0)
*30–39*	117 (54.2)
*40+*	32 (14.8)
Marital status	
*Single*	6 (2.8)
*Married*	200 (92.6)
*Cohabiting*	5 (2.3)
*Divorced*	5 (2.3)
Level of education	
*Primary*	4 (1.9)
*Secondary*	38 (17.6)
*Tertiary*	58 (26.9)
*Not stated*	116 (53.7)
Income	
*Low*	9 (4.2)
*Medium*	28 (13.0)
*High*	40 (18.5)
*Not stated*	139 (64.4)
Residence	
*Rural*	10 (4.6)
*Urban*	144 (66.7)
*Not stated*	62 (28.7)

Two thirds (144) of the participants had primary fertility (never conceived before) while three quarters (164) did not have living children. The overall period of infertility ranged from 1 to 21 years with an average of 5.6 ± 4.7 years whilst 98 (45.4%) of the couples had experienced 2–4 years of infertility and 94 (43.5%) had experience 5 or more years of infertility. About 1 in 5 of the participants had irregular menstrual cycles with 10 of them having experienced amenorrhoea of at least 1 year (Table 2).

**Table 2:** Reproductive characteristics

Characteristic	Frequency, n(%)
Number of pregnancies	
*Zero*	144 (66.7)
*One*	43 (19.9)
*Two*	14 (6.5)
*Three*	13 (6.0)
*Four*	2 (0.9)
Living children	
*Zero*	164 (75.9)
*One*	39 (18.1)
*Two or more*	13 (6.0)
Period of infertility (years)	
*1*	24 (11.1)
*2–4*	98 (45.4)
*5 and above*	94 (43.5)
Period in current relationship (years)^1^	
*1*	26 (12.0)
*2–4*	103 (47.7)
*5 and above*	87 (40.3)
Menstrual cycle	
*Regular*	158 (73.2)
*Irregular*	47 (21.8)
*Not stated*	11 (5.1)
Irregular cycle	
*Not defined*	15 (6.9)
*Infrequent*	22 (10.2)
*Amenorrhoea > 1 year*	10 (4.6)
*Not stated*	11 (5.1)
*Not applicable*^2^	158 (73.2)
Contraception	
*Nil*	170 (78.7)
*Coc*^*3*^	25 (11.6)
*Implant*	5 (2.3)
*Injectable*	7 (3.2)
*POP*^*3*^	3 (1.4)
*IUCD*^*3*^	4 (1.9)
*Condom*	2 (0.9)

^1^Period of infertility in current relationship

^2^Not applicable because they had regular cycles

^3^Coc = combined oral contraceptive pill; POP = progesterone only pill; IUCD = Intra-uterine contraceptive device

One hundred and thirty-nine (64.4%) of the participants had a known HIV status with 19(8.8%) being positive. Most (80.6%) of the participants did not have chronic medical conditions. Almost half of the participants (49%) were overweight or obese and 48 (22%) did not have a recorded weight (Table 3).

**Table 3:** Medical history and examination

Characteristic	Frequency, n(%)
HIV	
*Positive*	19 (8.8)
*Negative*	120 (55.6)
*Not stated*	77 (35.6)
Medical history	
*Nil*^*a*^	174 (80.6)
*Hpt/DM*^*b*^	17 (7.9)
*HIV*^*c*^	16 (7.4)
*Others*	9 (4.2)
Body mass index^d^	
*21–24/50–75*	58 (26.9)
*25–29/76–89*	60 (27.8)
*≥ 30/90*	46 (21.3)
*< 21/50*	4 (1.9)
*Not weighed*	48 (22.2)

^a^Nil means no recorded medical illness

^b^Hpt/DM means participant either had hypertension or diabetes mellitus or both

^c^Three of the HIV positive participants were recorded under Hpt/DM leaving 16 instead of 19 in this category

^d^Body Mass Index: Normal = BMI 21–24 kg/m^2^ or weight 50-75 kg; Overweight = BMI 25-29 kg/m^2^ or weight 76-89 kg; Obese = BMI ≥ 30 kg/m^2^ or weight ≥ 90 kg; Underweight = BMI < 21 kg/m^2^ or weight < 50 kg

**Fig. 1:**
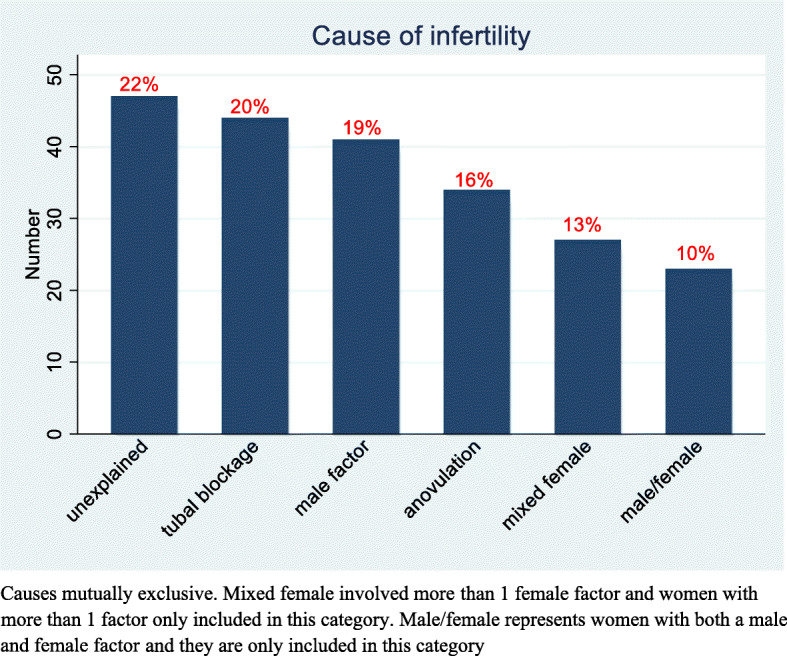
Causes of infertility

Only 97 (45%) women had their spouses undergo a semen analysis. The most common cause for infertility was ‘unexplained’ in 47(22%) of the women followed by tubal blockage affecting 44(20%), male factor [41(19%)], anovulation (34(16%)], mixed female factors (27(13%) and mixed male/female in 23(10%) (Fig. 1).

There was no association between person characteristics and cause of infertility (Table 4).

**Table 4:** Association between cause and person characteristics

Characteristic	Total	Cause						χ^2^ *p*-value
		Tubal blockage	Anovulation	Male factor	Male/ female	Mixed female	Unexplained	
Period of infertility (years)								
*1*	24 (11.1)	5	5	8	0	3	3	0.491^a^
*2–4*	98 (45.4)	20	13	15	12	12	26	
*5 and above*	94 (43.5)	19	16	18	11	12	18	
Age group (years)								
*< 30*	67 (31.0)	14	12	12	6	7	16	0.992^a^
*30–39*	117 (54.2)	22	18	22	13	17	25	
*40+*	32 (14.8)	8	4	7	4	3	6	
Any pregnancy								0.493
*Yes*	72 (33.3)	14	9	11	8	13	17	
*No*	144 (66.7)	30	25	30	15	40	30	
Any children								0.107
*Yes*	52 (24.1)	10	6	6	4	11	15	
*No*	164 (75.9)	34	28	35	19	16	32	

^a^Fisher’s exact p-value

A total of 49 (22.7%) women conceived/fell pregnant during the follow up period. Of these women who conceived 21(9.7%) had a live birth while 23 (10.7%) had an ongoing pregnancy at the end of follow up. Thirty-seven (17.1%) had ART in the form of IVF/ICSI or IUI. There was no association between cause of infertility and final outcome. ART was significantly associated with conception (*p* < 0.001 – fisher exact test) (supplementary Table 1).

Period of infertility and age group were significantly associated with conception (*p* = 0.006, *p* = 0.002 respectively) and in both cases it had a negative correlation, meaning less women conceived when the period of infertility became longer or with increasing age. There was no association between having had children and previous pregnancy with conception (supplementary Table 2).

## Discussion

This study of a largely urban population shows that 2/3 of the women had primary subfertility, an average period of infertility of 5.6 years. This average period encompasses the clinical definition of infertility (1 year), the epidemiological definition (2 years) and the demographic definition (5 years) meaning that most couples present when chances of natural spontaneous conception would have considerably waned off [4]. This is in agreement with a study amongst Sudanese couples where the mean duration was 4.9 years and 68.9% had primary subfertility [13] and with a study in Marrakech-Safi region of Morocco where about 2/3 of infertile couples had primary subfertility [14]. This is in sharp contrast to studies in Bauchi district of Nigeria where 38% (about 1/3) had primary subfertility [15] and in Erode were primary subfertility accounted for 90% of cases [15]. This difference in proportion of women with primary or secondary infertility in these countries is likely influenced by the causes of infertility peculiar to that region. Secondary subfertility tends to be higher in regions with high infectious morbidity such as tubal infections, post abortion and puerperal sepsis and hence likely low resource set ups. This study seems to have selected women in a higher socio-economic stratum by having a disproportionately higher number of participants from private gynaecologists. The subgroup of women mainly affected in this study was 30–39 years. This does suggest delayed presentation or failed intervention. Most of the participants tried to conceive with only one partner as shown by the similarity in period of total infertility and period of infertility in the current relationship (Table 2). This could mean that either infertility does not lead to increased divorce or that presenting for care selects out women in stable relationships in this population.

This study shows an almost equal contribution between tubal blockage (21%), male causes (19%) and unexplained (22%) infertility. This differs with the study in Bauchi where 27% of participants had tubal blockage, 18% had male causes and 12% were unexplained [11]. Another study in Erode India also showed a different profile of contributory causes with male causes being 26% and unexplained 6% [15]. Almost half of the causes are female factors constituted by tubal blockage, anovulation and a mixture of the two. This is in close agreement with the studies in Erode-India and Bauchi-Nigeria and Sudan which showed 45.5 and 51%and 49% female causes respectively [11, 13, 15]. This is higher than the contribution of female causes often quoted as 30–40% in the USA [5, 16]. Unexplained infertility was the biggest single cause of infertility in this study and is in agreement with the proportion found in other studies [4, 5]. This proportion is largely dependent on the thoroughness of investigations. The contribution of male factor infertility in this study can go up to 29% if we add the 10% who had both male and female causes. This falls within the range estimated for Sub-Saharan Africa in one systemic review [17] and in close agreement to studies in India and Sudan where male causes contributed to 26 and 36% respectively [13, 15]. The contribution of male causes is understated as only 97 (45%) women had their spouses undergo a semen analysis. This means that for women who had other causes, sperm dysfunction could also have been a co-existent cause. Hence the mixed male/female causes are likely understated. The study in Bauchi Nigeria had higher male participation (61.3%) [11]. This calls for more counselling to encourage male participation when screening for causes of infertility. Several studies have shown reluctance of male partners to participate in evaluation for infertility [18]. This is further compounded by cultural paternalistic beliefs which attribute infertility solely to female factors [17]. The most common cause of female infertility was tubal blockage even in women who had never conceived before. This in agreement with the study in Bauchi [11] but differs from studies in India [15] and Sudan [13] where PCOS and anovulation were the greatest contribution amongst female causes respectively. Tubal blockage infertility is mainly of infectious aetiology and can be prevented by early reproductive life interventions such safe sex and prompt treatment for pelvic infections. Rarely it is due to endometriosis which is suspected in women with dysmenorrhoea and pelvic pain. There is therefore need for community education to seek medical intervention early to prevent long term sequelae from these conditions. Anovulation presented a lesser burden than either tubal and male factor infertility. Almost half of the women who had recorded weights were overweight or obese and this is explained by the fact that the majority of participants were in the wealthier class as shown by their area or residency and level of education. This is almost similar to findings in the Bauchi district of Nigeria where 40% of the women were overweight or obese. This provides an opportunity to manage anovulation through weight control [5]. This calls for community interventions to curb the tide of obesity. Efforts must be taken to tackle obesogenic lifestyles which are characterised by limited physical activity and excess calories consumption.

There wasn’t much documented comorbidity in this population. The HIV prevalence in those with a known status was 13.7% which is in agreement with the HIV prevalence in the country of 12.7% [19]. This either means that infertility is not selecting out women with HIV or most women with HIV have their disease well controlled and hence not impacting on their fertility.

There was low access to ART in this study as is typical of low resource set ups [20].

The study provides the first ever calculation of the contribution of common aetiological factors to infertility in Zimbabwe. Its main limitation was failure to recruit an equal proportion of participants in private and public sectors. A larger number of patients in the public sector might have changed the proportion of causes of infertility and outcomes. It would also have allowed statistical comparison between the two groups. There was incompleteness of some of the retrospectively collected data especially the demographic variables as sometimes practitioners would not record these. This made it impossible to compare causes and outcomes against these variables. It also made it impossible to perform a multivariable analysis which would have given the independent association of each variable to causes of infertility and outcomes. The main strength was in assisting completion of investigations in some couples and hence allowing a diagnosis to be made. These results can only be generalised to an urban population where majority of patients afford private care. This is the case in many developing countries as governments rarely subsidise infertility treatments in the face of competing demands from high maternal morbidity and mortality [21]. This forces infertile couples to seek care in the private sector clinics.

## Conclusion

Most women present when chances of natural spontaneous conception are considerably reduced. This study shows an almost equal contribution between tubal blockage, male factor and unexplained infertility. Males are often not evaluated. Programs should target comprehensive assessment of both partners by encouraging male participation. Prevention and early treatment of sexual transmitted infections should remain a priority to reduce tubal factor infertility. Physical activity and low caloric intake should be encouraged to prevent obesity. Reproductive health programs must educate couples on the reduced chances of infertility with advancing age and the need to seek medical care early. Improved access to ART will result in improved pregnancy rates.

## Supplementary Information

**Additional file 1: Supplementary Table 1.** Cause of infertility and Final Outcome. **Supplementary Table 2.** Association between outcome and person characteristics.

## Data Availability

Data sets and materials available for sharing and uploading into a repository.

## Abbreviations

ART: Assisted Reproduction Technology
HIV: Human Immunodeficiency Virus
HSG: Hysterosalpingogram
ICSI: Intracytoplasmic Sperm Injection
IVF: Invitro-Fertilisation
IUI: Intra-Uterine Insemination
PCOS: Poly-Cystic Ovarian Syndrome
WHO: World Health Organisation
