# Supplementary material for: The causes of infertility in women presenting to gynaecology clinics in Harare, Zimbabwe; a cross sectional study
Source: Fertil Res Pract. 2021 Jan 5;7:1. doi: 10.1186/s40738-020-00093-0 (PMC7781825; doi:10.1186/s40738-020-00093-0)
Supplement: Supplementary file 1 — Additional file 1: Supplementary Table 1. Cause of infertility and Final Outcome. Supplementary Table 2. Association between outcome and person characteristics. [file 40738_2020_93_MOESM1_ESM.docx]

**Supplementary tables**

Supplementary table 1. Cause of infertility and Final Outcome

|  | **Outcome** | | | | | |  |  |
| --- | --- | --- | --- | --- | --- | --- | --- | --- |
|  |  | **not pregnant** | **pregnant** | **miscarriage** | **live birth** | **IUD** |  | **p-value** |
| **Diagnosis** | Tubal Blockage^1^ | 38 | 1 | 1 | 3 | 1 | 44(20.3) | 0.129^ϯ^ |
|  | Anovulation^2^ | 24 | 5 | 0 | 5 | 0 | 34(15.7) |  |
|  | male factor | 34 | 4 | 0 | 3 | 0 | 41(19) |  |
|  | male/female^3^ | 18 | 1 | 2 | 2 | 0 | 23(10.7) |  |
|  | mixed female^4^ | 17 | 7 | 1 | 2 | 0 | 27(12.5) |  |
|  | Unexplained^5^ | 36 | 5 | 0 | 6 | 0 | 47(21.8) |  |
|  | Total | 167(77.3) | 23(10.7) | 4(1.9) | 21(9.7) | 1(0.5) | 216(100) |  |
|  |  | **not pregnant** | **pregnant** | **miscarriage** | **live birth** | **IUD** |  | **p-value** |
| **Assisted Reproduction** | No ART^5^ | 148 | 18 | 2 | 10 | 1 | 179 | <0.001^ϯ^*** |
|  | Zim IVF^6^ | 10 | 5 | 2 | 5 | 0 | 22 |  |
|  | Other Country IVF | 4 | 0 | 0 | 3 | 0 | 7 |  |
|  | IUI | 5 | 0 | 0 | 3 | 0 | 8 |  |
|  | Total | 167(77.3) | 23(10.7) | 4(1.9) | 21(9.7) | 1(0.5) | 216(100) |  |

^ϯ^Fisher’s exact p-value, ***Significant at α=0.001

1. *This refers to blockage of both fallopian tubes as demonstrated by tubal patency tests*
2. *This refers to ovulatory function disorder as demonstrated by a diagnosis PCOS and low ovarian reserve*
3. *Mixed Male/female describes scenarios were both a male cause and a female cause was found. Such women were classified into this category only*
4. *Mixed female is when more than 1 female factor was present. They were only recorded in this category*
5. *No Assisted Reproduction technique offered*
6. *IVF done in Zimbabwe*

.

Supplementary table 2: Association between outcome and person characteristics

| Characteristic | Total | Outcome | | χ^2^ p-value |
| --- | --- | --- | --- | --- |
|  |  | Conceived | Not conceived |  |
| Period of infertility (years)  *1*  *2-4*  *5 and above* | 24(11.1)  112(51.9)  80(37.0) | 9(37.5)  28(28.6)  12(12.8) | 15(62.5)  70(71.4)  82(87.2) | 0.006** |
| Age group (years)  *<30*  *30-39*  *40+* | 67(31.0)  117(54.2)  32(14.8) | 24(35.8)  23(19.7)  2(6.3) | 43(64.2)  94(80.3)  30(93.8) | 0.002** |
| Any children  *Yes*  *No* | 52(24.1)  164(75.9) | 14(26.9)  35(21.3) | 38(73.1)  129(78.7) | 0.402 |
| Pregnancy  *Yes*  *No* | 72(33.3)  144(66.7) | 19(26.4)  30(20.8) | 53(73.6)  114(79.2) | 0.358 |

**Significant at α=0.01
